# Synthetic, Natural, and Semisynthetic Polymer Carriers for Controlled Nitric Oxide Release in Dermal Applications: A Review

**DOI:** 10.3390/polym13050760

**Published:** 2021-02-28

**Authors:** Carolina Gutierrez Cisneros, Veerle Bloemen, Arn Mignon

**Affiliations:** 1 Surface and Interface Engineered Materials, Campus Group T, KU Leuven, Andreas Vesaliusstraat 13, 3000 Leuven, Belgium; Caro.Cisneros@KULeuven.be (C.G.C.); Veerle.Bloemen@KULeuven.be (V.B.); 2 Prometheus, Division of Skeletal Tissue Engineering, KU Leuven, Herestraat 49, 3000 Leuven, Belgium

**Keywords:** NO-donor, topical release, polymeric matrices, microbial infections, wound healing, blood circulation, semisynthetic polymers

## Abstract

Nitric oxide (NO•) is a free radical gas, produced in the human body to regulate physiological processes, such as inflammatory and immune responses. It is required for skin health; therefore, a lack of NO• is known to cause or worsen skin conditions related to three biomedical applications— infection treatment, injury healing, and blood circulation. Therefore, research on its topical release has been increasing for the last two decades. The storage and delivery of nitric oxide in physiological conditions to compensate for its deficiency is achieved through pharmacological compounds called NO-donors. These are further incorporated into scaffolds to enhance therapeutic treatment. A wide range of polymeric scaffolds has been developed and tested for this purpose. Hence, this review aims to give a detailed overview of the natural, synthetic, and semisynthetic polymeric matrices that have been evaluated for antimicrobial, wound healing, and circulatory dermal applications. These matrices have already set a solid foundation in nitric oxide release and their future perspective is headed toward an enhanced controlled release by novel functionalized semisynthetic polymer carriers and co-delivery synergetic platforms. Finally, further clinical tests on patients with the targeted condition will hopefully enable the eventual commercialization of these systems.

## 1. Introduction

Nitric oxide is a free radical molecule that is produced endogenously in the human body. Its chemical properties make it suitable for the regulation of several physiological processes, including circulatory, immune, neurological, and antioxidant responses [1]. Given its widespread participation in biological systems, inadequate amounts of it (both deficiency and overproduction) can result in illness [2]. While an excess of nitric oxide is implicated in hypersensitive responses and neurodegenerative disorders, its lack results in cardiovascular and neurological complications, hypertension, sexual dysfunction, etc., especially for patients with a pre-existing condition such as diabetes [3,4].

This has brought special interest in the generation of biomedical strategies that carry and deliver nitric oxide, or its precursors, exogenously. Being a gaseous free radical, it has a short half-life in vivo; hence, the main challenges for its administration in the clinic are stable storage and controlled release. This has led to the blending of nitric oxide donors with carriers. Numerous of these platforms have been developed where polymeric scaffolds are often used [3]. Their physicochemical properties as well as their loading and release capacities have been studied and improved in the course of the past 20 years.

Polymers are a strong pillar of drug delivery technologies given that they can offer intrinsic therapeutic activity, be biodegradable to prevent accumulation or toxicity, and enhance release kinetics. Moreover, their molecular structure can be engineered for more biocompatibility and strict control over a wide range of drugs. This tunable release can be in constant doses over long periods, cycled, or condition-responsive, all of which can improve the therapeutic value of the system [5]. This is by the enhancement of molecule transport and because they can become active participants in the treatment. For instance, their biocompatibility can contribute to increased stability for cellular uptake [3]. The inherent properties of the materials vary according to their origin, while synthetic polymers such as poly(ethylene oxide) (PEO) and poly(vinyl alcohol) (PVA) are characterized by their reproducibility and desirable mechanical properties, natural polymers including chitosan, alginate, and gelatin, are valuable due to their biocompatibility, biodegradability, mucoadhesion and antibacterial capacity, for instance [6].

Considerable advances in polymer synthesis have contributed to the availability of a wide range of polymeric scaffolds with different architectures (multi-layered, compartmentalized, branched, etc.). These platforms have advanced regarding physical properties by particle entrapment, with the possibility to tune the capsule size and concerning chemical advances that allow for surface functionalization, all of which contribute to controlled release kinetics [7]. Further improvement in polymer carrier systems has been addressed by the introduction of semisynthetic polymers, where both natural and synthetic polymers are linked to each other to obtain a combination of their properties. Along with the evolution of polymers, the therapeutic effect of drug co-delivery by different release triggers is another increasing trend that has accomplished synergistic treatments.

Dermal (cutaneous) administration of NO-donors has been increasingly studied as a promising therapy for related skin conditions. The endogenous synthesis of nitric oxide in human skin can be either enzymatic or chemical and it is required for various processes such as pigmentation, blood flow dynamics, cutaneous tissue regeneration, and skin immune response [3,8]. The focus of this review consists of the topical NO-delivery by polymer matrices according to their application target, with a special highlight on the nature of the applied polymers. For a better understanding of these platforms, an overview of the biochemistry and the dermatological role of nitric oxide, and the resulting disorders is given in the upcoming sections (Section 2, Section 3, and Section 4). Afterward, nitric oxide donors and their possible carriage systems are explained (Section 5 and Section 6) before reviewing the pharmaceutical dermal applications in detail (Section 7). Finally, remarks are made on the future perspectives of these technologies (Section 8).

## 2. Biochemistry of Nitric Oxide

Nitrogen monoxide, or nitric oxide (NO•), is a colorless, free-radical gas molecule. It is non-combustible but as a hydrogen bond acceptor, it can react with oxygen. The particular molecular characteristics and reactivity make this reactive oxygen species relevant in biological systems. In water, it has poor solubility and reactivity, but it has a high diffusion rate. Given that it complexes metals, it participates in corporal catalyzes by coordination with enzymatic units [9]. The bioabsorption of NO• is based on its high solubility in hydrophobic solvents as it can cross membranes, lipidic biological barriers, without the need for active transport. Given that such molecules with unpaired electrons are rare in the human metabolism, the reactivity of NO• is selective, which is a useful property to moderate biological systems [10,11,12].

Nitric oxide’s reactivity with oxygen gas is due to an unpaired electron in the anti-bonding π molecular orbital between the nitrogen and oxygen atoms of the molecule, which needs another unpaired electron to stabilize. Oxygen gas is unpaired in the ground state because its electrons, despite being an even amount, occupy different molecular orbitals. This extensively studied reaction is still not fully understood, yet the currently accepted reaction scheme consists of the following two equations [10,13]:

(1) $$2\mathrm{NO}↔N_{2}O_{2}$$

(2) $$N_{2}O_{2}+\mathrm{NO}\to N_{2}O+{\mathrm{NO}}_{2}$$

Compared to other radical molecules, NO• is considered relatively stable because, in oxygen-free conditions, it can only dimerize at low temperatures or high pressures. The conditions for this are not fulfilled anatomically; thus, this reaction does not risk taking place in the human body. However, at high aerobic conditions, NO• is quickly oxidized into reactive nitrogen species, from which dinitrogen trioxide (N_2_O_3_) predominates in aqueous systems, such as that of the human body [14]. N_2_O_3_ is an unstable molecule, so it is quickly metabolized by hydrolysis [10,14,15].

The chemical properties of nitric oxide allow it to have a cell messenger role, which specifically contributes to the regulation of corporal responses by various signaling networks. The behavior of NO• under physiological conditions accounts for its participation in a wide variety of biological responses. This is further addressed in the following section [10,16].

## 3. Nitric Oxide in Human Physiology

NO• is present in the human body in a concentration range in the order of pM to nM and it is used for regulatory functions [10]. The metabolic synthesis of NO• results from the enzymatically controlled oxidation of L-arginine, an essential alpha-amino acid involved in protein synthesis. Nitric oxide synthases (NOS) are the enzymes that regulate this process; they have two redox regions—an electron generating carbon-terminal (reductase) and a nitrogen-terminal (oxidase). NOS synthetize one molecule of L-arginine into one molecule of NO• and stoichiometric amounts of L-citrulline as a byproduct [15,17,18].

There are two classes of NOS. The first types are constitutive, calcium-regulated, cNOS. When activated for a physiological function, they produce low amounts of nitric oxide. The activation is transient because it follows the signal’s kinetics in a pulsed form. The second types are inducible NOS (iNOS), which are regulated genetically and transcribe for immune and inflammatory responses. They are continuously active once induced, for instance, by bacterial lipopolysaccharides or cytokines. There is a delay of hours for the production of NO• due to the time for protein synthesis, but the reached NO• concentration is higher and sustains for as long as the enzyme is present [10,15,19].

In addition to the enzymatic release, nitric oxide can be physiologically generated by the chemical reduction of nitrate (NO_3_) or nitrite (NO_2_). For metabolism, NO_3_ is converted by commensal bacteria to NO_2_ [20]. Sweat is a nitrate source for the body and given a higher amount of sudoriparous glands in the skin, higher NO• amounts are found there [17,21]. The present review focuses on the dermal application of nitric oxide; hence, the following subsection focuses on metabolic activity at a cutaneous level.

### Dermatological Nitric Oxide

The skin comprises around 6% of the total body weight. Its outermost layer is the epidermis, a dense avascular epithelial structure that protects organs; it mainly consists of keratinocytes. The next skin layer is the dermis, which is a mucous polysaccharide matrix that contains encapsulated collagen and elastic fibrils, where metabolic exchange happens. Within this structure, different functions take place that are regulated by NO•, including the synthesis of connective tissue, the immune and inflammatory responses, and the pigmentation of the skin. The deepest skin layer is the hypodermis, a subcutaneous lipidic layer whose blood and lymphatic vessel networks respond to nitric oxygen radicals, for the formation of new blood vessels (angiogenesis). Finally, sudoriparous glands and hair follicles originate at a subcutaneous level and disperse in human skin. Sudoriparous glands produce sweat, which is an indirect source of NO• [21].

Both classes of NOS enzymes described earlier in Section 3 are needed for dermal homeostasis. There are three bioregulatory roles of NO• in human skin—vasodilation, cutaneous immune response, and tissue regeneration. The first is predominantly regulated by low cNOS synthesis. It consists of vascular smooth muscle relaxation following induction by endothelial cells of skin vessels. Its deficiency results in the contraction of vessels that narrows the arteries. When this happens, blood cannot flow properly and it causes circulatory conditions such as ischemia [4,22]. The second role is the immune response against environmental threats. Injury protection from UV-light or pathogens is provided by high NO• production. This can be either enzymatically activated by iNOS, or in an enzyme-independent manner from the metabolism of sweat’s nitrates. Nitric oxide present on the skin is cytotoxic for a wide variety of pathogens; therefore, its chemical production on the body surface prevents and clears infections [22,23]. Finally, nitric oxide has an important role in skin regeneration by modulating inflammation, cell proliferation, extracellular matrix deposition, and angiogenesis. All three dermal NO-regulated processes contribute to wound healing [4,22,23,24,25,26].

Triggered for defense and repair, most skin cell types can synthetize NO• by one or more NOS for inflammatory, antimicrobial, and apoptotic responses. Another stimulant is exposure to UV, i.e., sunlight, which activates iNOS for skin pigmentation. Skin pigmentation occurs in the dermis and epidermis by melanocyte cells. Nitric oxide contributes to the regulation of this process as a response to UV-light exposure. Negative feedback is carried out indirectly by growth factors and directly by NO• molecules [8,10,14,21,27,28,29].

Under pathophysiological conditions, nitric oxide can be overproduced, due to increased immune and inflammatory responses; or underproduced by inhibition. The unsuitable presence of NO• in the body results in circulatory disorders regarding blood activity and vessels. Furthermore, some enzymatic antagonists of NOS have been proven to damage circulation locally [4,22].

## 4. Nitric Oxide Skin Disorders

Given that both excess and a lack of NO• lead to pathological states, fine control of NOS expression and NO• production is needed. Excessive production of nitric oxide promotes oxidative stress, causing degenerative diseases. Large amounts of NO• associate with undesirable infectious or inflammatory responses. NO• can induce toxic reactions against other host tissues, especially in particular types of inflammation, such as asthma. The role of NO• in allergen-induced skin inflammation is pro-inflammatory at low concentrations, while it induces inflammatory cell apoptosis at high concentrations. Elevated iNOS expression has been reported in skin conditions such as psoriasis, dermatitis, and atrophy. The extracellular matrix deposition, collagen synthesis, and cytokinetic promotion of angiogenesis, overstimulated by NO• may result in abnormal scarring by disproportionate (hypertrophy) or fibrous (keloid) tissue [14,24,25,26,28].

However, decreased skin NO• levels also cause impairment, of which the first consequence is endothelium dysfunction. This condition is related to atherosclerosis, high blood pressure, and other factors that may lead to cardiac events and disorders related to angiogenesis. Insulin resistance is associated with nitric oxide deactivation by dietary oxidation. At low availability of L-arginine, endothelial NOS can generate superoxide, which scavenges nitric oxide. This has been reported in pathological conditions such as diabetes and hypercholesterolemia. Furthermore, insufficiency of nitric oxide results in wound healing incapacity. This is especially problematic for patients that suffer from diabetes, malnutrition, or chronic steroid treatment. Sepsis patients present decreased NO• production, linked to a decreased and inadequate production of arginine. Undernourished infants also present arginine deficiency, which is similarly manifested by lowered nitric oxide production [8,10,15,26,30,31,32,33].

Because insufficient nitric oxide bioproduction results in a vast number of pathological conditions, at the end of the 19th century, sodium nitroprusside, glyceryl trinitrate, and isoamyl nitrite were used to treat the deficiency for the first time, although their physiological effects were not completely known. These pharmacologically active substances that carry nitric oxide are called NO-donors. Ideally, the donors stabilize the radical until the release is required [33,34].

## 5. Nitric Oxide Donors

Nitric oxide is an inconvenient molecule to handle in gas form due to long treatment time by impractical and expensive equipment for delivery (a gas tank with a diluting system). There is an additional concern about the presence of toxic concentrations for host cells [35,36]. Therefore, NO-donors have been designed as substances by which the radicals can be steadily delivered in situ [37]. Given that NO• is a radical of high therapeutic relevance, systems that improve their deliverability have been developed. Despite the simplicity of the molecule, its exogenous application is challenging due to the complexity and manifold of reactions, tissue specificity, and concentration dependence [8,34,37].

**Figure 1:**
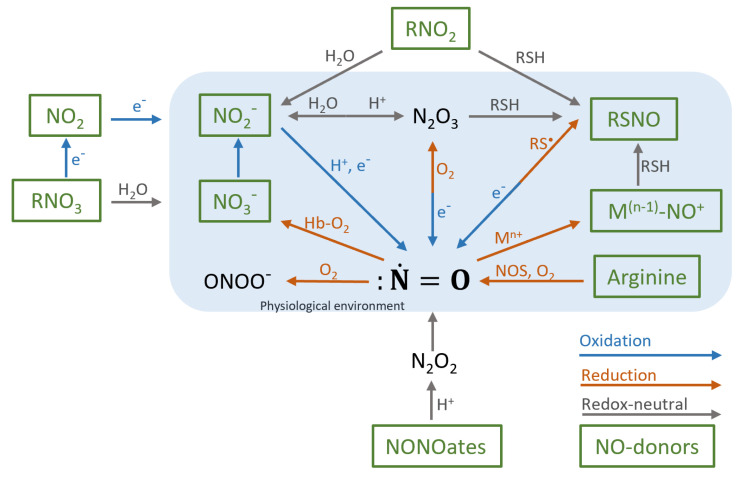
Physiological production of nitric oxide starting from different NO-donors. This figure was adapted from [33]. Abbreviations: hemoglobin (Hb).

The biochemical pathways where nitric oxide is involved are the fundament for the design of NO-donors. There are eight molecules that can be metabolized into nitric oxide and that can be used as donors in biological systems (see Figure 1). These consist of arginine, metal nitrosyl complexes (M^(n−1)^-NO^+^), N-diazeniumdiolates (NONOates), nitrate ion (NO_3_^−^), nitrogen dioxide (NO_2_), nitrite ion (dioxidonitrate(1-), NO_2_^−^), nitrosothiols (RSNO), organic nitrite (RNO_2_) and organic nitrate (RNO_3_) [33]. They can be classified as natural or chemical-occurring, according to their physiological presence.

Natural NO-donors occur biologically and include arginine, metallonitrosyl complexes (M^(n−1)^-NO^+^), NO_2_^−^, NO_3_^−^ and RSNO. Assuming that NOS are present and active, L-arginine can be provided to increase NO• production. However, this can often not be affirmed; hence, enzyme-independent donors are needed. Nitric oxide binds to transition metals to form metallonitrosyl complexes. These are endogenously formed at a physiological level when NO• binds metallic centers of enzymatic units. Their usefulness as NO-donor suppliers relies on their photosensitivity to decompose to nitric oxide, which can be used for controlled release [38]. While NO_2_^−^ can be directly metabolized to nitric oxide, NO_3_^−^ has to be reduced to NO_2_^−^ first. This conversion is performed by bacterial enzymes that are present in the human body. If not converted, NO_3_^−^ remains stable to flow through the circulatory system. Finally, nitrosothiols require an electron to decompose into nitric oxide and thiolate (RS–).

**Scheme 1:**
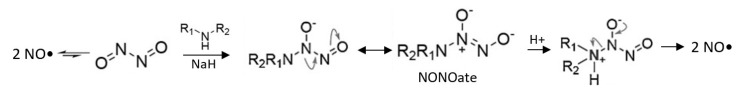
Formation and degradation scheme of N-diazeniumdiolates (NONOates). This scheme was adapted from [33].

Chemically occurring NO-donors do not exist physiologically, but they are viable donors because they can be metabolized by the human body. These are NO_2_, RNO_3_, RNO_2,_ and NONOates. Provision of nitric oxide by NO_2_ requires its reduction to a nitrite ion. Organic nitrate can either be reduced to nitrogen dioxide or be hydrolyzed to nitrate ion. Organic nitrite is transnitrosated into a nitrite ion by water or into RSNO by a thiol (RSH). NONOates are one of the most frequently used NO-donors. Upon protonation to dinitrogen dioxide (N_2_O_2_), the dimerized form of nitric oxide (which does not occur in physiological conditions), provides two NO• radicals as shown in Scheme 1. NONOates are N-based analogs of diazeniumdiolates; their production consists of the reaction of nitric oxide gas with primary or secondary amines [33].

### 5.1. Controlled Release

Ideally, the nitric oxide delivery profile and release location must be precisely controlled, which can be achieved by a triggering substrate. As the first possibility, NONOates have been designed to respond to substrate modulation. Their spontaneous breakdown can be prevented, for instance, by an alkylation that can later be selectively removed by a particular molecule or enzyme. This technology is inspired by fluorescence biosensing chemistry [33].

The second possibility is to trigger NO• release photonically. This is convenient, given that light is adaptable, non-invasive, and adjustable. Sometimes, NO-donors are spontaneously photosensitive; therefore, unless this pathway is disabled, they cannot serve for controlled release. Usually, the alternative is to deactivate NONOates with a photo-labile group. To monitor the localization of the donors microscopically and to control the release of NO•, fluorescence emission has been used [33].

The variety of NO-donor structures available (summarized in Table 1) results in different reactivity and delivery kinetics. This can happen either spontaneously, enzymatically, or chemically. S-nitrosothiols and diazeniumdiolates, for instance, release it through an autolytic breakdown triggered by thermal or photonic factors. For an enzymatic source, either prodrugs or mimetics are used. Prodrugs are immobilized enzymes in biomaterials characteristic of a controlled bioconversion and mimetics consist of endogenous activation by non-enzymatic compounds [34,37].

### 5.2. Main NO-Donors

The most frequently studied and implemented NO-donors are S-nitrosothiols (RNSO) and NONOates [41]. The discovery of S-nitrosothiols (RSNO) as endogenous NO-donors encouraged the development of exogenous for the delivery of NO• [42]. On the other hand, diazeniumdiolates (NONOates) are the most widespread NO-donors given that they do not need to be redox-active for release because they imitate nitric oxide biosynthesis. They consist of structures with a functional NONO-group [1].

RSNO synthesis is carried-out in liquid solute by S-nitrosation of thiols. Some of the RSNO that result from this reaction are S-nitrosoglutathione (GSNO), S-nitroso cysteine (CySNO), S-nitroso-N-acetylcysteine (SNAC) (depicted in Table 1), S-nitrosoalbumin, and S-nitrosohemoglobin. GSNO is the most widely used RSNO in research because it can easily be purified by precipitation and drying, which makes it relatively stable for storage. On the contrary, the use of CysNO or SNAC implies their fresh preparation upon application. S-nitrosoalbumin and S-nitrosohemoglobin are less used in general, due to their instability in solution. RSNO are prone to photochemical decomposition so they require light protection. Another broadly studied synthetic RSNO, characterized by high thermal stability, is S-nitroso-N-acetylpenicillamine (SNAP). It is found commercially but cannot be integrated into hydrogels due to its low water solubility. Furthermore, it has been avoided in topical formulations to eliminate the possibility of undesired effects by excessive concentration in the hypodermis due to its high hydrophobicity [42].

NONOates are convenient NO-donors because of their biocompatibility, as they only release the initial amines from which they were made upon physiological decomposition to nitric oxide. Furthermore, they are stable in solid state and only decompose when dissolved in a biological environment [1]. The release is spontaneous, but it can be controlled by amine groups contained in a polymer matrix, which can also be convenient to avoid unwanted leakage [43]. Some common NONOates are diethylamine NONOate (DEA/NO), spermine NONOate (SPER/NO), and 1-(hydroxy-NNO-azoxy)-L-proline (PROLI/NO) (see Table 1) [40].

Despite the broad spectrum of NO-donors, only a few have been approved and are available commercially. This may be due to the amount of testing for approval and to the fact that these therapeutic applications have only been recently developed. Commercially used NO-donors are used for a particular, clinically proven application such as nitroglycerine for acute angina and Nitropress for heart failure and high blood pressure [33,34].

## 6. Carriers of Nitric Oxide Donors

For dermal applications, carriers are often used for localized release. There are several possibilities to contain NO-donors, such as in dendrimers, micelles, liposomes, polymer particles, and metalorganic crystals. An innovative approach to harness the beneficial properties of NO• is to attach a releasing moiety to an existing drug. Different hybrid compounds can offer various drug actions with synergistic effects, with reduced toxicity and side effects [34,37]. For dermatological application, in particular, there are three main classes of carriers—liposomes, metals, and polymers, which are discussed in further detail in the following subsections.

### 6.1. Liposomal Carriers

Liposomes are spherical vesicles made of bilayers of phospholipids and cholesterol; they are amphiphilic: hydrophobic at the outside while hydrophilic at the inside. Comparable to NO• biochemistry, liposomes can easily penetrate the epidermis due to their lipidic composition. This effect is also used to control the kinetics and physicochemical features together with the choice of the NO-donor [23,34].

The encapsulation of NO-donors inside liposomes results in sustained release because their decomposition is prevented by the lipidic bilayer coating. Some of the recent developments in liposomes as NO-donor platforms include the design of photoinduced NO-releasing liposomes by the incorporation of Ru salen, a metal photosensitive nitrosyl complex [44]. Attachment of fluorescent immunoassays on and inside liposomes has also been reported as it can be used for monitoring of NO• in time and location (spatiotemporal) [33,45,46]. Liposomes are biodegradable and biocompatible; therefore, research in this area is promising. However, they are hydrophobic, which limits their cutaneous application due to excessive accumulation in lipidic membranes [23,34].

### 6.2. Metallic Carriers

Metallic particles are highly tunable, and they have been studied extensively. Silver and gold are especially popular metals for skin application because of their antibacterial and anti-inflammatory properties, in addition to their functional modification possibilities. The capacity to prevent infection of NO• is enhanced by these metallic platforms [23].

A popular metallic carrier platform is nanoparticles. Their fabrication and surface functionalization is versatile for controlled release. The size and location of nanoparticles within the system control the kinetics by the probability of contact with water to release the NO-donors. Small particle size is directly proportional to the bactericidal effect, while the sphericity is inversely proportional. These nanoparticles range in size from 1 nm to 100 nm with convenient optical and electrical properties. For instance, functionalized gold nanoparticles with grafted NONOate-forming polymers have allowed for their proton-induced delivery [47]. Despite the steady progress of such therapeutic technology, the challenge for their medical use is still the possible toxic effect of bioaccumulation and the control over membrane crossing [46].

Another modern platform consists of metal–organic crystalline structures, they are metallic centers with bound organic linkers resulting in porous materials with a high surface area. Their properties are adjusted according to the combination of linker and metal. Because NO• can be adsorbed within the pores, they are pharmaceutically useful as NO-donor delivery systems. The release happens upon contact with water but it can be controlled. For instance, highly regulated, photon activated NO-donor delivery by nitroimidazole bound to zinc has been reported. The release varies according to the duration and intensity of the irradiation [48]. The photoactivation can also be monitored and induced by fluorescence [34]. This technology is based on covalent bond coordination with polymers, which is the following class of NO-donor carrier addressed.

### 6.3. Polymeric Carriers

The advantage of polymers for dermal-controlled delivery of NO• resides in the possibility of adjusting the physicochemical properties. These donors can be either synthetic or natural-based polymers or a combination of both. Synthetic polymers are well understood and convenient, especially for their mechanical properties. Synthetic poly(lactic-co-glycolic acid) (PLGA) nanoparticles, for example, are FDA approved for their use as drug carriers and they have been widely used due to their sustainable release properties. Interest in natural ones has increased in the last years because of the therapeutic properties of the carrier itself. An example is chitosan, displaying anti-inflammatory and antibacterial properties with sustainable, controlled drug release [23,49]. A combined type of polymer carriers has emerged in recent years with the intention of joining the advantages of synthetic and natural polymers, the so-called semisynthetic polymers. Different types of NO-donors have been encapsulated in these molecules via technologies such as emulsion polymerization, with control on their molecular mass and functional ends to adjust the profile of delivery [34,50].

The first described polymer carrier matrices were hydrogels; they are currently still used to entrap nanoparticles. This type of system is desirable due to optimizable polymer particle size, solubility, and release kinetics [51]. Alternative polymeric matrices consist of micelles and dendrimers, dissolved in a buffer or gel for topical applications. Self-assembled micelles are used to encapsulate NO-donors due to their amphiphilic nature with a hydrophobic core. Additionally, moieties can be adhered for a controlled release. Dendrimers are globular molecules produced by branching polymers with attached reactive groups. Their size and shape can be chemically tuned; they have been functionalized with NO• moieties to produce NONOates with a significant antimicrobial capacity [52].

Biomedical applications that make use of polymeric carriers exploit their specific properties being their molecular size, porosity, solubility, complexation, bioavailability, toxicity, cellular and molecular effects, etc. Currently, interdisciplinary research develops biocompatible and biodegradable drug delivery platforms made of synthetic, natural, and semisynthetic polymers to treat cutaneous inflammation, dysfunction, and infection. They seek to enhance the mechanical and physical characteristics of these materials regarding the efficiency of release [53]. There are multiple pharmacological factors that affect this, which result in a wide spectrum of properties. These factors include the polymer type, drug loading, polymer breakdown mechanisms, interactions between the drug and polymer, etc. [51].

Synthetic polymers have often superior mechanical properties compared to natural polymers, and they are abundantly present and are easier to process into suitable pore sizes and scaffold geometries than natural ones. Natural polymers do not have the robustness of the design of synthetic polymers, but their biocompatibility makes them stand out. Natural polymers are often more effective in physiological conditions than synthetic polymers because, in most cases, they are biodegradable in the human body. Important drawbacks they have are their limited purity and high molar mass dispersity, resulting in variability of the characteristics, which is unacceptable for the regulated environment of the pharmaceutical industry [54]. Therefore, laborious purification and analytical testing are needed to ensure high quality. Ideally, semisynthetic polymers succeed to preserve the desirable properties of synthetic polymers From a chemical point of view, semisynthetic polymers enrich the field of drug-carrying scaffolds by optimizing physicochemical properties for a targeted delivery [51].

The investigation and optimization of mechanical and physical material characteristics of novel polymer carriers is still the focus of drug delivery, as will be seen in the upcoming sections of this review. Strict regulation of the surface properties can prevent possible toxicity to human cells by controlling NO• delivery [34]. The uses of polymer matrices for cutaneous delivery of NO-donors are the object of this review and they are addressed according to their pharmaceutical applications in the following section.

#### Polymer Carrier Preparation

The carrier preparation technologies have advanced alongside the evolution of polymers. In the beginning, most materials were directly solvent cast with the simultaneous entrapment of NO-donors [55]. This allowed the loading of sufficient concentrations for therapeutic effects according to the NO-donor of choice (often in the millimolar range), but there was no control of the release given its spontaneous delivery in physiological conditions [56,57]. There was an initial burst in the first five minutes, where most of the loaded drug would be released [58]. Given that solvent casting is the simplest and the least controlled preparation, lyophilization and electrospinning were introduced to achieve higher quality and control of the material and their processing [6,52,59,60].

Simultaneously, polymer carrier nanoparticles began to be tested according to their size, porosity, and surface charges to achieve high NO-donor encapsulation efficiencies. Nitric oxide release from these nanoparticles depends on different mechanisms that have been measured and modeled, including desorption, diffusion, particle erosion, or a combination of them. Kinetic measurements have demonstrated sustained NO release for up to ca. 24 h after an initial peak [60,61,62].

In the case of modern polymers, highly controlled chemistry technologies were necessary for the preparation of nanoparticles, which were either directly tested or entrapped in hydrogels and electrospun mats. These implemented technologies were also necessary for applications where the NO-donor was grafted into the polymer, which is another possibility to extend and control the period of release. They include light-mediated, polymerization-induced self-assembly and reversible addition–fragmentation chain transfer [63,64].

## 7. Pharmaceutical Applications

The clinical applicability of NO-donors is impeded by a lack of control regarding the release rate and target location. Therefore, their delivery through carriers has enabled the proposal of various pharmaceutical applications. Research has focused on the employment against bacteria and biofilms, but they have also been increasingly investigated for their use in wound healing enhancement and in the treatment of circulatory conditions [34]. The evolution of each category is discussed based on an assemblage of pertinent publications.

### 7.1. Antimicrobial Applications

NO• is an antimicrobial agent whose activity is dependent on the concentration—low production by cNOS stimulates the immune system by regulation of the immune cell cycle, while iNOS, as part of the innate immune system, produce larger quantities that respond to bacterial polysaccharides and endotoxins, and pro-inflammatory cytokines [8]. When nitric oxide began to be used to treat skin infections in the late 1900s, it was mainly applied in a gaseous state or by nitrite provision to supply NO• as an alternative treatment to antibiotic-resistant microorganisms such as *C. albicans* bacteria and *Leishmania* parasites. It was also initially tested for particular skin conditions such as psoriasis [65,66,67,68]. However, the need for controlled and localized release and more convenient platforms shifted the development of nitric oxide supply to NO-donor carriers.

In the beginning, these platforms were tested using silane and zeolite. With the purpose to treat infections, silane particles were conjugated with NO-donors. Alternatively, these were carried by zeolites given that they are porous crystal minerals with high compatibility and storage capacity [69]. By 2000, interest in polymeric platforms began to rise significantly to achieve a steady prolonged release of NO•. Polymers are versatile materials, capable of delivering constant, sustained doses of drugs of which the dosage may be tuned and cycled. Advances in polymer technology have focused on the development of biocompatible degradable responsive systems with higher permeation and retention capacities [5]. In Table 2, a summary of publications on infection-treating NO-carrying polymeric formulations for dermal applications dating from 2003 until 2020 is presented (this is further discussed in more detail in the following paragraphs).

#### 7.1.1. Synthetic Polymer Carriers

Until 2010, only synthetic polymers were used to carry nitric oxide. These synthetic hydrogels have been made of materials including poly(vinyl alcohol) (PVA), poly(vinyl pyrrolidone), poly(urethane) (PU), Pluronic F-127 (PL), and (hydroxyethyl)methacrylate [55,56,57,58,80,81]. PU is used for its semi-permeability, i.e., impermeable to bacteria but permeable to vapor and air [82]. The main NO-donor used was GSNO, a well-known commercial nitrosothiol. Given that the microbicidal capacity of NO• was known, an early development was the incorporation of two different nitrosothiols—GSNO and SNAC into hydrogels made of Pluronic F-127. These were tested for NO-release by irradiation as potential topical therapeutics. They highlighted that a hydrogel matrix damps the initial rate of thermal NO release compared to aqueous solutions [57].

Other recurrent precursors of nitric oxide are organic and inorganic nitrites. Jones et al. reported the use of such a precursor in a nitric oxide-releasing chamber made of a gas-permeable acrylic polymer. Inside the chamber, the dismutation of inorganic sodium nitrite salt (NaNO_2_) resulted in the sustained release of NO• during the fermentation of immobilized glucose by lactic acid probiotics (*L. fermentum*) [79]. In 2008, another delivery system based on NaNO_2_ was published by Friedman et al. The platform consisted of glass-state disaccharide films (trehalose and sucrose) within which NaNO_2_ was chemically reduced to generate and trap nitric oxide using lyophilization. The resulting material was incorporated in a composite hydrogel matrix with poly(ethylene glycol) (PEG). The NO-release profile varied in function of the PEG composition and they achieved a steady state level of nitric oxide that could be maintained for at least 24 h [81]. The system’s release was sustained and tunable; therefore, it was tested against methicillin-resistant *Staphylococcus aureus* (MRSA) in 2009 by the optical density of bacterial cultures, histochemistry of cultured tissue, and histological processing of the skin of a mouse model [80]. In 2017 [54] and 2018 [73], the platform was tested again to treat reference strains of a dermatophytic fungus *Trichophyton rubrum*. These NO-releasing polymer matrices were assessed as a more effective alternative for current treatments, which face antifungal resistance, side effects, and insufficient penetration [76]. In all cases, the technology effectively reduced the microbial abscesses and proved promising for the intended therapeutic application. In 2018, the in vitro test consisted of the measurement of inhibition of 40 fungi compared to a standardized treatment. After 24 h of exposure, it resulted in 99.9% of fungal reduction [73].

Regarding the evaluation of the NO-releasing hydrogels, in the beginning, only the physicochemical properties and NO-release profile were studied, their bactericidal effect in vitro was not characterized yet. However, for the first time in 2004, GSNO-containing PEO hydrogels were tested on the skin of healthy human subjects and found to deliver nitric oxide sustainably [56]. Five years later, a successful antibacterial test against *P. aeruginosa* with a photoactive, NO-releasing platform was published by Halpenny et al. [58]. The system consisted of a PU hydrogel containing metal-NO complexes of Mn and Ru. This was also the first time that combined antimicrobial agents were tested. Additional to the NO-donor, two other active substances were included, i.e., hydrogen peroxide and methylene blue, a phenothiazinium dye with reported anti-MRSA activity. The light-controlled co-delivery proved more effective against the treated colonies than the separate effect of the agents. In the same year, Han et al. applied their NO-releasing hydrogel on cutaneous infections of a murine model to prove that the application of topical nitric oxide reduced the bacterial burden of MRSA [80].

In addition to testing the antibacterial efficacy, two publications in 2010 reported efficacy against other pathogens such as fungi and parasites. The first publication was the earlier explained probiotic chamber of Jones et al. [79], but in this system, it was tested for efficacy against bacteria (*E. coli*, *S. aureus*, *P. aeruginosa*, *A. baumannii*, and MRSA) and two pathogenic fungal strains (*T. mentagrophytes* and *T. rubrum*) [79]. In all cases, the eradication capacity was confirmed under sustained therapeutic levels of released nitric oxide. The second publication by López-Jaramillo et al. described a randomized clinical trial in which 178 patients suffering cutaneous leishmaniasis, a parasitic skin infection, were treated. The tested therapy consisted of acidified organic nitrite encapsulated during the electrospinning of thermoplastic PU hydrogels. Even though the results showed more than twice less effectiveness than the prevalent medication (meglumine antimoniate), the use of NO-donors remained an alternative for treatment given that adverse effects were greatly reduced [78]. At this point, the in vitro testing for antibacterial properties of NO-donors became a usual practice but this did not ensure the platforms’ safety for their clinical use. For this purpose, in vivo experimentation and clinical trials were needed.

#### 7.1.2. Natural Polymer Carriers

By 2011, the proof-of-concept of polymeric NO-donor systems was achieved, so efforts to improve the polymer carrier system were starting. Given the desire for suitable functional groups for medical applications and the need for toxicity reduction, natural polymers were introduced as NO-donor carriers. The manipulation and the mechanical versatility of synthetic polymers allow the production of materials that fit a particular biological application. However, they are not biodegradable, which means that the resulting byproducts may not be able to be discarded renally [83].

Therefore, natural polymers from hydroxyacids were implemented. They have molecular structures whose characteristics cannot be achieved in synthetic systems. The biodegradability, for instance, by enzymatic hydrolysis is an attractive property of natural polymers as toxic byproducts are avoided. These byproducts are already part of the human metabolic system. Natural carriers also have potential bioactivity, which could make them active contributors to the treatment [84]. The first natural-based polymer carrier for cutaneous NO-donor delivery consisted of chitosan and alginate combinations used to encapsulate GSNO in nanoparticles. They observed that NO• was released spontaneously in physiological conditions with an initial burst, which was tested for cytotoxicity against fibroblasts to ensure their safety for their application onto mammalian cells [59].

Two more publications using the same natural carriers were reported in 2013 and 2014. The first one consisted of a very similar nanoparticle system to the one discussed in the previous paragraph [59], with the difference that instead of direct GSNO encapsulation, glutathione, a GSNO precursor, was encapsulated in the chitosan/alginate mucoadhesive polymers where it was nitrosated to then release NO in a sustained manner. The delivery was carefully studied against a varying pH and temperature and the nanoparticles were tested with two cytotoxicity tests. The parameters were compared to the free release of GSNO. Its encapsulation in natural biodegradable, non-toxic nanoparticles resulted in a more stable release profile with complete removal of cytotoxicity [6]. The second publication consisted of the same natural polymers, where the encapsulation of a different nitrosothiol (S-nitroso-mercaptosuccinic acid) was tested against *S. aureus* and *E. coli* bacteria. The results showed a positive correlation between NO-delivery and its antibacterial effect [60].

Several years later, in 2020, three purely natural polymer-based platforms were reported. Two articles by Urzedo et al. reported the evaluation of alginate nanoparticles where the encapsulated NO-donor was S-nitroso-mercaptosuccinic acid. The antibacterial tests performed were against three bacterial strains—*E. coli*, *S. mutans,* and *S. aureus*. They compared the antibacterial effect of the pure nanoparticles to the co-release of biogenic-produced silver nanoparticles, which are antimicrobial as well. They reported that the biodegradable and biocompatible nanopolymers were highly bactericidal while non-toxic to mammalian cells. The co-delivery proved to have a synergistic effect by an increased antibacterial capacity [70,71].

Banerjee et al. tested a cellulose topical gel in a randomized clinical trial. The formulations contained different doses of the berdazimer sodium (NVN1000) NO-donor, a macromolecular polysiloxane polymer that stably stores NO• as a covalently bound N-diazeniumdiolate [85]. These formulations are commercially available under SB206 by Novan Inc. It was applied on 256 patients for the treatment of *M. contagiosum*, which is a viral infection [86]. The results revealed significant clearing of the lesions with minimal adverse effects after 12 weeks of daily application. The best performing dose was the highest (12% NVN1000) regarding the optimized balance between antiviral and side effects during the course of the study [72].

Natural polymers exhibit different chain sizes according to monomer ratios. This inherent variation is undesirable to obtain consistent materials. Another important shortcoming is compromised stability due to poor mechanical properties and high water solubility. Therefore, blending and crosslinking with other materials to enhance these properties has been researched [83]. Furthermore, although pure natural polymer delivery platforms have been often proven non-cytotoxic at high concentrations, the different combinations must also be carefully tested to ensure their safe application on biological systems. Since the introduction of natural polymers as nitric oxide carriers, they have been prepared by the ionic gelation method regardless of the presentation of the carrier [70,71].

#### 7.1.3. Semisynthetic Polymer Carriers

By 2018, a new type of material was used as NO-donor carriers: semisynthetic polymers. They consist of the combination of natural polymers with synthetic polymers by blending, grafting, or crosslinking. The outcome is a material that combines the desirable mechanical and thermal strength of synthetic polymers, and they often are still biodegradable and as biocompatible as natural polymers. Thus, semisynthetic polymers can potentially overcome the limiting properties of purely synthetic and purely natural carriers [50]. Therefore, an increasing tendency in the last years to combine natural and synthetic polymers to optimize the particle properties is also observed in Table 2.

For instance, chitosan nanoparticles, containing S-nitrosothiol (RSNO) as NO-donor, incorporated into Pluronic F-127 hydrogels have been dermally tested in murine models. The incorporation of these systems has proven to profit the advantages of both polymers in terms of physicochemical and biological properties; hence, sustained NO• release for bactericidal concentrations while remaining harmless for mammalian cells [2,75].

The efficacy of additional semisynthetic polymer nanoparticles was reported in the same year by Stasko et al. The tested molecules made of hexylene glycol and cellulose, loaded with NONOate were applied to treat different cutaneous fungal infections, including *Trichophyton*, *Epidermophyton*, *Fusarium*, *Candida*, and *Malassezia* species. The gel formulation containing berdazimer sodium was characterized regarding NO• release, and it was reported to effectively clear the infected tissue from bacteria [74].

#### 7.1.4. Further Enhancement of Polymer Carriers

In order to enhance the release profile and control of synthetic polymers, there has been growing interest in the direct functionalization of NO-donor into the polymer backbones. This has been achieved by highly controlled polymerization conditions and modern polymer chemistry technologies such as reversible addition–fragmentation chain transfer, a quasi-living radical polymerization. This approach is a very reliable one for tailor-made structures with reduced variability by decreasing the distribution range of molecular mass [83]. In 2016, NONOate-functionalized dendrimers were produced by the modification of poly(amidoamine) (PAMAM). PAMAM dendrimers are highly branched macromolecules synthetized into a particular structure. This is achieved by the addition of defined generations of methyl acrylate and ethylenediamine in an ethylenediamine core [87]. The utility of this system is the functionalization capacity of the superficial branches by the NONOate group. The dendrimers coupled with the NONOate were entrapped in electrospun PU fibers and they resulted effective in the inhibition of *P. aeruginosa*, *E. coli*, *S. aureus,* and MRSA, while not being cytotoxic in vitro [52].

In 2017, a second functionalized polymer carrier was published. Poly(oligoethylene) glycol methyl ether methacrylate (POEGMA) nanoparticles, produced by polymerization induced self-assembly, were functionalized with diazeniumdiolate to release NO• upon contact with water. The measured release was extended compared to the free NO-donors by limited access to water within the polymeric capsules [62]. In 2018, an oligoethylene glycol-based polymer was also functionalized with NONOate to release NO•gas; this was reported effective against *P. aeruginosa* biofilms. In this research, the polymer, an amphiphilic statistical ternary copolymer, was a carrier and also designed to be actively antimicrobial by cell membrane disruption [64]. Statistical copolymers are those in which the monomer distribution obeys established statistical laws. This can result from alternation, clustering, or no arrangement tendency [88]. One year later, Shen et al. reported polyethylene oxide micelles they functionalized with N-nitrosoamine to release NO• with a photoinduced control due to photon-cleaved N–NO bonds, and coumarin chromophore as a visual reporter for monitoring. These amphiphilic structures were loaded with the antibiotic ciprofloxacin in the core for more efficient bacterial eradication through synergistic co-delivery [63].

As mentioned earlier, co-delivery is another approach to enhance the therapeutic capacity of NO-releasing platforms. In the course of the last five years, developments on nanopolymeric NO-carrier synergistic systems have been made. An early publication of a combination of hydrogen peroxide and methylene blue into the NO-donor platform was reported in 2009 and detailed in Section 7.1.1. [58]. Another co-delivery was shown by Nguyen et al. in 2016 and proven to be more effective than the separated treatments. Their POEGMA nanoparticles carrying acetonitrile NONOate and gentamycin antibiotic were tested against *P. aeruginosa* infection and a decrease of more than 90% of the viability was measured [61].

NO-donors have also been compared to available therapies separately. For instance, Sulemankhil et al. assessed the antimicrobial activity of NO• gas, released by enzymatic reaction of at a sustained rate during 18 h. The technology was proven to be more effective than other antimicrobial dressings containing iodine, silver, and antibiotics (chlorhexidine, gentamicin, and vancomycin) against three clinically relevant antibiotic-resistant micro-organisms—*A. baumannii*, methicillin-resistant *S. aureus*, and *P. aeruginosa* [77].

Even if these composite and highly technological platforms hold a great potential application as NO-delivery platforms, exhaustive experimentation and clinical trials are required to ensure the safety and efficiency of the systems to eventually translate them to the medical field.

### 7.2. Wound Healing Applications

The role of NO• in wound healing includes its antimicrobial capacity, but it also has an effect on the stimulation of cytokine production, on the regulation of growth factor expression, and on the deposition of the extracellular matrix. Catalytic nitric oxide production is critical for wound healing, as the fluid in the lesion induces NOS in different cell types.

Wounds that fail to heal in form and type are called chronic. Chronic wounds are an ailment in multiple conditions, which could be related to decreased nitric oxide levels. In diabetes, for instance, insufficient production has been reported, related to a reduced inflammatory response. Malnutrition and radiotherapy-induced injury are additional conditions associated with inadequate healing. Finally, steroidal drugs, to treat conditions such as asthma and arthritis, strongly damage the healing process by altering arginine’s metabolism. Both L-arginine supplementation and NO-donors can potentially restore such impairments [8,22,89].

On the contrary, in some conditions where the skin lesions are hypersensitive, such as in the case of psoriatic patients, the reduction of NO• production may be needed. One way to achieve this is by the suppression of enzymatic pathways to control the lesions’ excessive angiogenesis [8,65].

The wound healing application of NO-donors has been increasingly tested once the research on the antimicrobial potential of similar systems had begun. This is probably explained by the fact that for wound healing applications, protection against microbes is only one of the functions of nitric oxide to be tested. Therefore, the platforms promoting wound healing correspond to more recent investigations, which are compiled chronologically in Table 3. In general, the target condition to be treated with the NO-donor was the improvement of the wound healing process in terms of time for regeneration and quality of the new tissue. However, additional complications have also been tested, including infection, diabetes, ischemia, and acne.

Despite the encouraging results of the discussed platforms, the treatment of chronic wounds remains a challenge. For this reason, research has focused on improving the polymer carrier technologies in terms of stability, biodegradability, and controlled release to make them more suitable for skin repair. Additionally, NO-releasing wound dressings have been tested in combination with additional compounds such as anti-inflammatory agents to enhance the healing capacity.

#### 7.2.1. Synthetic Polymer Carriers

In comparison to the evolution of platforms intended for microbial applications, the same trend with a delay can be seen in wound healing applications. For instance, initially, several purely synthetic polymer carriers were evaluated. Initially, Pluronic F-127 was a frequent polymer used to contain GSNO nitrosothiols in hydrogel matrices. Specifically, three publications based on the same platform were published in 2007 [109], 2008 [108], and 2010 [106]. The latter study focused particularly on impaired wound healing of patients with an ischemic condition. All three articles confirmed amelioration in the healing process by increased collagen deposition and tissue granulation, which resulted in earlier closure and re-epithelialization of the wound [106]. It was highlighted that the hydrogel application worked best when applied in the initial phases of re-epithelialization, i.e., proliferation and inflammation [108,109].

In 2009, the technology that was developed by Friedman et al. and published in 2008 (previously described in Section 7.1.1.), was used in a nanoparticle matrix to treat MRSA-infected wounds. Acceleration of the closure of infected wounds and antimicrobial properties confirmed that the system was effective in the dermal treatment of infected wounds [107]. Another unmodified synthetic polymer hydrogel matrix was published in 2018 by Hlaing et al. They tested for improvement of healing in wounds infected with MRSA through a PLGA emulsion with entrapped GSNO. They confirmed improvement in wound healing in vivo and confirmed the antibacterial capacity both in vitro and in vivo [99]. Finally, in 2020, a GSNO-incorporated PEG-gel was tested in vitro and in vivo on MRSA-infected wounds by Lee et al. [92].

Synthetic polymer carriers have performed outstandingly as carriers of NO-donors for wound healing applications. However, hydrogels made of commonly used non-hydrolyzable synthetic polymers, such as PVA and Pluronic-F127, demonstrate insufficient loading capacity and do not stimulate bioabsorption of the carried drug. This is undesirable for NO-delivery. Another shortcoming is the risk of harmful degradation products. To reduce these limitations, different natural polymers and polymer blends have been developed [41].

#### 7.2.2. Natural Polymer Carriers

In the case of wound healing applications, the first natural-based carrier was published in 2013, while this was already reported by 2011 in antimicrobial applications. Polymer carriers of purely natural origin have been tested for wound healing since then as biocompatible and biodegradable alternatives. A stronger tendency to use polymers of proteic nature (i.e., gelatin and collagen) can be identified, in contrast to those intended to treat infections that are mostly based on polysaccharides. This can be explained by the fact that those natural polymers need to be actively synthetized during re-epithelialization (i.e., collagen deposition); therefore, they offer an additional benefit by actively helping to accelerate the healing process.

In 2013, an enzyme-controlled platform was developed by Gao et al. They prepared a naphthalene-capped short polymer gel composed of phenylalanine and glycine amino acids in a 2:3 ratio. β-galactoside-caged NO• was incorporated in the gel to regulate the release by the cleavage activity of β-galactosidase. Testing in vivo proved that controlled release of nitric oxide-induced angiogenesis and resulted in faster wound healing. Another natural-polymer platform was published in 2015, it consisted of a chitosan film with GSNO to treat infected wounds with two bacterial strains, *P. aeruginosa* and *S. aureus*, which were particularly selected for having different cell membrane compositions. Interestingly, the films showed an effective reduction of both bacterial strains, as opposed to the application of pure chitosan films, where a decrease in viability was measured due to the polymer’s antibacterial properties. The results also proved an enhancement of healing by accelerated inflammation and tissue granulation [103].

Five years later, in 2020, three more platforms were published with a natural-based wound healing hydrogel as a carrier, namely by Nie et al. [91], Póvoa et al. [95], and Huang et al. [94]. The first system was designed to treat diabetic wounds based on a cellulose gel containing nitric oxide and asiaticoside, an anti-inflammatory plant extract. The results proved that the platform adequately adhered to the skin and promoted healing of diabetic skin ulcers by up-regulation of Wnt/β-Catenin signaling pathway, which activates iNOS [91]. The second matrix was a “self-expandable” sponge produced by lyophilization of collagen with GSNO. They reported accelerated healing by an effectively bioabsorbable platform [95].

The last system was developed to treat infected wounds with gelatin methacrylamide hydrogels. Hyaluronic acid was added due to its cell repulsive properties to avoid excessive adhesion. The NO-donor consisted of bis-N-nitroso compound 6 (BNN6, Bis(2-methoxyethyl)nitrosoamine). These hydrogels were light-responsive due to the presence of graphene oxide nanosheets functionalized with β-cyclodextrin and they were tested in two bacterial strains—*E. coli* and *S. aureus*—by in vitro and in vivo experiments [94].

The results demonstrated enhanced healing of wounds infected with bacteria [94]. However, lack of control and premature release of NO• has been measured due to instability of the NO-donors in heterogeneous, insufficiently strong natural carriers. Furthermore, certain natural polymers such as proteins promote cell adhesion, which in excess may be counterproductive as a support matrix during the treatment at the clinical level [83].

#### 7.2.3. Semisynthetic Polymer Carriers

The combination of synthetic and natural polymers to enhance the properties of the separate carriers was reported earlier in wound healing applications (2012) than in antimicrobial applications (2018). This can be explained by the fact that in wound healing, the predominating matrices are biocompatible natural hydrogels, for which the enhancement of mechanical properties may be of higher priority than for the anti-microbial applications (where gel or spray administration alternatives are sufficient). In 2012, Jones et al., who also published the probiotic chamber in 2010 (detailed in Section 7.1.1. [79]), developed another chamber containing *L. fermentum* for the in-situ production of nitric oxide. In this study, the patch was made of a combination of alginate and a synthetic acrylic polymer and they proved that the healing of ischemic and infected dermal wounds in a rabbit model was accelerated [105].

Five years later, another semisynthetic platform was published by Zhou et al. [35]. They developed a poly(ε-caprolactone) (PCL)/chitosan electrospun hydrogel, where glycosylated diazeniumdiolates were grafted onto the side chain of chitosan. Given the formation of a CS–NO bond, the release of nitric oxide was enzymatically controlled by glycosidase. This technology was tested on ischemic chronic wounds and demonstrated enhanced healing by pro-angiogenesis, immunomodulation, and enhanced collagen synthesis. Finally, PU/gelatin/keratin electrospun hydrogels were produced in 2020 by Wan et al. [93]. In this development, further enhancement was achieved by N-nitrosation of keratin to form a coupled nitrosothiol NO-donor. Their composite system released nitric oxide with no cytotoxic effect to mammalian cells and it promoted skin repair by cell proliferation and a decreased inflammatory response. It also demonstrated an antibacterial effect against *E. coli*. These platforms feature functionalization, which is a strategy to improve polymer carriers, addressed in detail in the next section.

#### 7.2.4. Further Enhancement of Polymer Carriers

In the attempt to improve the release properties of polymer carriers for the treatment of wounds by NO-donors, the active molecules have been functionally coupled into polymer chains in around 50% of the wound healing applications (Table 3). Additionally, synergistic additives have also been applied in order to enhance the tissue regeneration capacity of the treatment. In 2002 already, an exceptionally elaborate application was published by Bohl et al. They tested an aminated-PVA hydrogel functionalized by covalent attachment of NO• to the polymer backbone. The design was meant to treat chronic wounds of diabetic patients. Thus, it was tested in vivo in mice with induced diabetes. They clearly observed that the nitric oxide provided actively participated in the wound healing process by enhancement of the tissue quality, although the exact mechanisms of action were not yet known. They hypothesized it was related to the genetic regulation of growth factors and collagen synthesis [110].

NO-functionalized polymer hydrogels were reported in 2015 [101,102], 2016 [100], 2019 [96,98], and 2020 [90]. An acrylonitrile-functionalized vinyl terpolymer, with enhanced mechanical properties, was electrospun with a defined fiber-morphology, to control NO-release. The electrospun mats were tested in vivo [102]. Another system consisted of Pluronic F-127/PVA hydrogels, where PVA was chemically functionalized by esterification with mercaptosuccinic acid of SNO groups, with a subsequent S-nitrosation to release nitric oxide. The polymer combination was biologically tested in vivo only for the acceleration of wound healing [101]. Wound closure was also promoted in vivo by diazeniumdiolate-coupled propylene oxide mixed with poly(ethylene imine) (PEI) in a PEG-gel [96].

Particular wound conditions have also been targeted by research. For instance, NVN1000 covalent-functionalized polysiloxane was tested by Baldwin et al. in a clinical trial in 153 subjects as a therapy for acne vulgaris (lesions infected by *P. acnes*) [100]. It was found that the daily application successfully decreased the amount and size of wounds and it was well tolerated. Another tested condition was MRSA-infection of diabetic wounds; the system consisted of PEI/PLGA nanoparticles where PLGA was functionalized with NONOate. The results of in vivo, ex vivo, and in vitro testing were positively correlated with antibacterial and wound healing capacities [98]. PLGA nanoparticles were also conjugated to treat MRSA-infected wounds in 2020; however, the NO-donor, in this case, was GSNO [90].

In most research regarding wound healing, the experimental testing follows a general structure. The NO-delivering therapy is produced and tested for NO• release. In the case of a novel carrier, its physicochemical characterization is performed first. For instance, electrospun functionalized dressings have been characterized by techniques including attenuated total reflection-Fourier transform infrared spectroscopy for the verification of functional coupling of the NO-donor to the polymer carrier; this is verified with scanning electron microscopy by visualizing the morphology of the fibers [35,93]. In the case of hydrogel matrices, further physicochemical characterization has been often carried-out to obtain parameters that describe the swelling capacity, degradation rate, rheological properties, and compression strength [94]. When nanoparticles are incorporated into these platforms, they are carefully characterized separately. Relevant parameters for this form of carriers include size, polydispersity, sphericity, and electric potential, which are measured by dynamic light scattering [98,99].

In Table 3, it can be observed that almost all the wound healing research so far has been pre-clinical, i.e., in vivo testing in murine or rat models [101]. The wound assessment is quantified in terms of inflammation, wound discharge, granulation tissue, induration, skin maceration, color, etc. [102]. Additional complications of wound healing because of the treatment also require further testing. For instance, those studies focusing on infected wounds also performed in vitro tests to confirm the antibacterial capacity.

Finally, cytotoxicity for mammalian cells is also often tested in vitro for an initial safety test of the presented systems. Despite in vitro and in vivo testing, in addition to some clinical trials that have also been successfully conducted, it is not enough to achieve the introduction of these platforms to the clinic. Approval of molecular entities and platforms by the FDA is particularly strict; therefore, the extrapolation of in vitro and in vivo experimentation requires substantial funding and collaborations between the clinical and pharmacological research [111]. This translation takes time and NO-donor therapeutics are still a relatively new field.

### 7.3. Circulatory Applications

Given NO-donors’ ability to activate hemodynamics by increased vasodilation and blood flow while reducing the aggregation risk, they stimulate research in therapeutic targets to treat related chronic and debilitating conditions [4]. This regulatory function of nitric oxide is less applicable at a cutaneous level (excluding its need during skin regeneration addressed in Section 7.2), but it is interesting for systemic delivery such as in the case of biomedical implants. The reason for this is that NO-donors for cutaneous applications (such as creams and gels) have low water solubility, while they are highly soluble in hydrophobic matrices. Accordingly, the released molecules can reach the smallest blood vessels and potentially lead to uncontrolled systemic vasodilation with undesirable side effects [11]. Because of this risk, safety is to be ensured in dermal NO-donors in circulatory applications by ensuring a localized, controlled release. In some studies, it has been assessed how the dermal release of NO• regulates circulation and these studies are addressed in the present section.

An early topical vasodilatory application was reported by Souto et al. in 2010. The unconventional application of a nitric oxide-releasing gel consisted in therapy for women with sexual disfunction by the enhancement of clitoral blood flow. The gel was made of Pluronic F-127 (PL) and it carried GSNO nitrosothiol as NO-donor. The measurement of clitoral arteries by Doppler ultrasound demonstrated that desired local vasodilation without systemic effects was achieved within 15 min of its application in 20 healthy, sexually active women [112]. Hence, this formulation can be of use to treat women’s sexual disfunction if proven in a larger sample size, where subjects with the ongoing sexual disorder also participate.

Another topical NO-release platform for skin vasodilation was published by Vercelino et al. in 2013, as an alternative treatment of painful inflammation medically known as nociception. For the production of hydrogels, they made use of PL to carry GSNO, the same components as Souto et al. (see the previous paragraph). The hydrogels were tested in vivo in rats with induced hypernociception and healthy human volunteers. The platform resulted in a local increase of dermal blood flow as a potential analgesic (pain-relieving) dermal therapy of inflammation. Again, the limitation in this study was the sample size (n = 5) [11].

Circulatory applications at a cutaneous level were no longer reported for the following seven years, until 2020, when Soares et al. tested a commercial nitroglycerin patch for the treatment of microvascular dysfunction of ischemic patients [113]. Nitroglycerin patches date from 1985, and they are commercially available to treat chest pain, medically called angina pectoris [114]. Soares et al. applied Mylan-Nitro patches by Mylan Pharmaceuticals, consisting of silicone-coated polyester films in form of a pouch where nitroglycerin is contained [115]. The study was carried out to investigate if the placement of a NO-donor locally could serve as microvascular protector by dilation of brachial arteries. This was confirmed in 10 healthy men [113]. The same year, a similar application was recommended in a review published by Appleton et al., where they examined different trials on the potential capacity of stroke prevention by nitroglycerin NO-donor patches administered dermally. Their conclusion was to recommend the treatment given its safety proven by a meta-analysis. However, this was only ensured as a preventive mechanism. Thus, it is not yet safe to use in ultra-acute strokes [116].

A novel NO-donor hydrogel has been reported in 2020 as a microvascular therapeutic. The technology consists of a nitrite-functionalized PVA patch produced by reversible addition−fragmentation chain-transfer polymerization of nitrosothiols. Giglio et al. tested the platform in vivo in healthy volunteers as a proof-of-concept and determined a significant increase in blood circulation with potential application in microvascular diseases [117].

In most of the circulatory applications of NO-donor polymeric carrier systems healthy volunteers were invited for an initial stage, but in vivo tests still need to be performed on people suffering from each one of these diseases.

## 8. Future Perspectives

Plentiful polymer-based platforms have been designed, characterized, and tested as carriers of NO-donors for therapeutic applications. Nitric oxide is a radical gas species that participates in the regulation of several cutaneous processes. In topical applications, its half-life is brief. For an efficient and functional NO• delivery in biomedical applications, control of the release is needed. A recurrent problem of the available technologies is the unrestrained scattering of nitric oxide or leaching of byproducts produced during the release. Some platforms have aimed to solve these issues by the use of semisynthetic polymers, whose byproducts are often safe, or by the control of release through functionalization or caging the NO-donor. However, even if a more controlled release has been achieved, the developed systems tend to be complicated or not safe yet for clinical use, given a risk of interactions, cell toxicity, or other unknown side effects.

Novel NO-delivery carriers for topical therapies are still to be developed for targeted release. Some recent materials have been proposed as promising alternatives. For instance, PCL is a polymer that has been blended more recently for cutaneous application of nitric oxide as it is ductile and bioabsorbable. It has been reported to provide desirable mechanical properties and degradation kinetics [41]. Finally, a novel Laponite nanoclay has been proposed as a NO-donor carrier because it can potentially prevent leaching and release controlled amounts of NO• [118]. The development and implementation of these carriers could eventually offset the current challenges of NO-releasing platforms. This could stimulate their clinical testing for approval for administration to patients.

Since 1867, nitric oxide therapy has been the subject of pharmacological approaches because it was identified as a powerful cutaneous regulator of vasodilation, immune response, and skin regeneration. However, it is not absent of side effects such as generating tolerance or undesired oxidative stress [119]. Numerous conditions are related to the reduced bioavailability of NO•, but even if the administration of NO-donors has shown positive evidence from animal studies, more translational studies are required for their implementation in the clinical field.

## Figures and Tables

**Table 1:** Representative NO-donors according to chemical class, adjusted from [33].

NO-donor.	Representative Examples		Trigger for NO• Generation	
	Structure	Name	Non-Enzymatic	Enzymatic
Organic nitrate	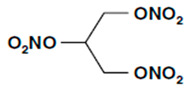	GTN [33]	Thiols	Cyt-P450, GST, etc.
Organic nitrite	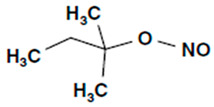	Tert-pentyl nitrite [33]	Hydrolysis, trans-nitrosation, thiols, light, heat	Xanthine oxidase
Metal–NO complex	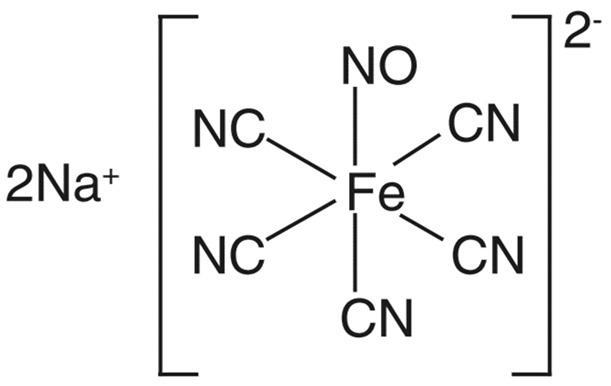	Sodium nitroprusside [38]	Light, thiols, reductants, nucleophiles	Membrane-bound enzyme
Nitrosothiol	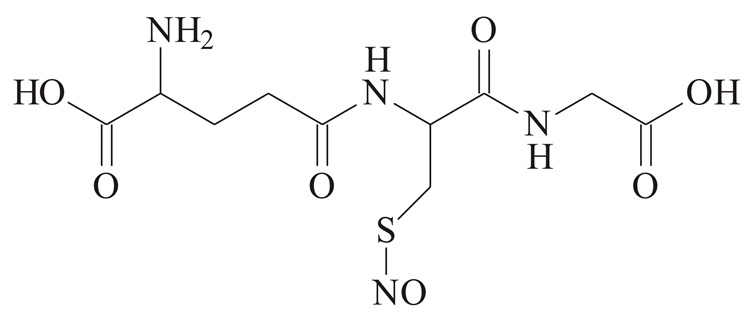	GSNO [39]	Spontaneous, enhanced by thiols, light, metal ions	Unknown enzymes
	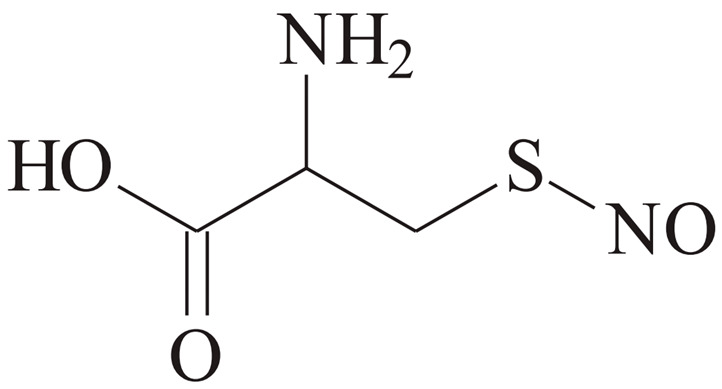	CySNO [39]		
	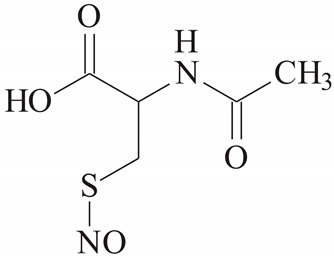	SNAC [39]		
NONOate	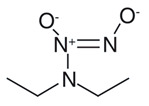	DEA/NO [40]	Spontaneous, engineered to be light or pH-responsive	Caged for control by β-galactosidase
	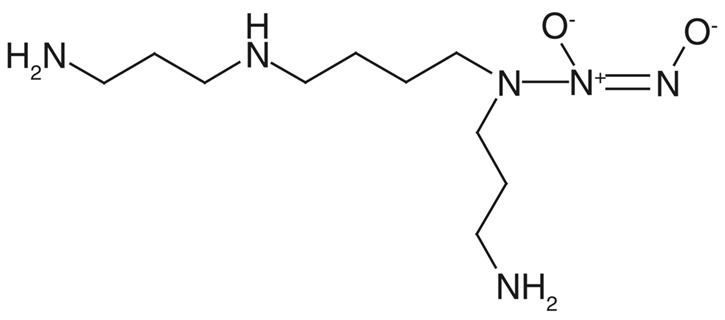	SPER/NO [40]		

Abbreviations: Nitroglycerin (GTN), S-nitrosoglutathione (GSNO), S-nitroso cysteine (CySNO), S-nitroso-N-acetylcysteine (SNAC), diethylamine NONOate (DEA/NO), spermine NONOate (SPER/NO).

**Table 2:** Compilation of publications showing the evolution of NO-donor polymeric carriers intended to treat dermal infections.

Year	Origin	Carrier	Matrix	NO-Donor	Donor Coupling	Synergistic Additive	Release Trigger	Antimicrobial Test	Clinical Trial	Ref.
2020	N	Alg	np	Nitrosothiol	-	Silver np	-	Bacterial	-	[70]
2020	N	Alginate	np	Nitrosothiol	-	Silver np	-	Bacterial	-	[71]
2020	N	Cellulose	Hydrogel	NONOate	-	-	-	Fungal	Yes	[72]
2019	S	PEO	Micelles	N-nitrosamine	Yes	-	Light	Bacterial	-	[63]
2018	S	PEO	Hydrogel	NO• gas	Yes	-	-	Bacterial	-	[64]
2018	S	PEG/Disaccharide	np	Inorganic nitrite	-	Antifungal	-	Fungal	-	[73]
2018	SS	PEO/Cellulose	np	NONOate	-	-	-	Fungal	Yes	[74]
2018	SS	Chitosan/PL	np	Nitrosothiol	-		-	-	-	[2]
2018	SS	Chitosan/PL	Hydrogel	Nitrosothiol	-		-	Bacterial	-	[75]
2017	S	PEG/Disaccharide	Hydrogel	Inorganic nitrite	-	-	-	Fungal	-	[76]
2017	S	POEGMA	np	NONOate	Yes		-	Bacterial	-	[62]
2016	S	PU/PAMAM	Dendrimers	NONOate	Yes	-	-	Bacterial	-	[52]
2016	S	POEGMA	np	NONOate	-	Antibiotic	-	Bacterial	-	[61]
2014	N	Alginate/Chitosan	Capsules	Nitrosothiol	-	-	-	Bacterial	-	[60]
2013	N	Alginate/Chitosan	np	Nitrosothiol	-	-	-	-	-	[6]
2012	S	PEO	Chamber	NO• gas	-	-	Enzyme	Bacterial	-	[77]
2011	N	Alginate/Chitosan	np	Nitrosothiol	-	-	-	-	-	[59]
2010	S	PU	Hydrogel	Organic nitrite	-	-	-	Parasitic	Yes	[78]
2010	S	Acrylic polymer	Chamber	Inorganic nitrite	-	-	Bacteria	Bacterial, fungal	-	[79]
2009	S	PEG/Disaccharide	Hydrogel	Inorganic nitrite	-	-	-	Bacterial	-	[80]
2009	S	PU	Hydrogel	Metal-NO complex	-	H_2_O_2_, methyl-ene blue	Light	Bacterial	-	[58]
2008	S	PEG/Disaccharide	Hydrogel	Inorganic nitrite	-	-	-	-	-	[81]
2004	S	PEO	Hydrogel	Nitrosothiol	-	-	-	-	Yes	[56]
2004	S	PVA/PVP	Hydrogel	Nitrosothiol	-	-	-	-	-	[55]
2003	S	PL	Hydrogel	Nitrosothiol	-	-	Light	-	-	[57]

Abbreviations: Polymer origin: natural (N), semi synthetic (SS), synthetic (S). Polymer carriers: poly(ethylene oxide) (PEO), pol(ethylene glycol) (PEG), Pluronic F-127 (PL), poly(oligoethylene glycol) methyl ether methacrylate (POEGMA), poly(urethane) (PU), poly(amidoamine) (PAMAM), poly(vinyl alcohol) (PVA), poly(vinyl pyrrolidone) (PVP). Matrix: nanoparticles (np). NO-donor: diazeniumdiolate (NONOate).

**Table 3:** Compilation of publications showing the evolution of NO-donor polymeric carriers intended to treat cutaneous wounds.

Year	Origin	Carrier	Matrix	NO-Donor	Coupled Donor	Synergistic Additive	Trigger for Release	Antibacterial Test	Trial	Extra Target Condition	Ref.
2020	S	PLGA	np	Nitrosothiol	Yes	-	-	Yes	in vivo	Infection	[90]
2020	N	Cellulose	Hydrogel	NO• gas	-	Plant extract	-	Yes	in vivo	Diabetic skin ulcer	[91]
2020	S	PEG	Hydrogel	Nitrosothiol	Yes	-	-	Yes	in vivo, in vitro	Infection	[92]
2020	SS	PU, gelatin, keratin	Hydrogel	Nitrosothiol	Yes	-	-	Yes	in vivo, in vitro	-	[93]
2020	N	GelMA	Hydrogel	N-nitrosamine	Yes	Hyaluronic acid	Photons	Yes	in vivo, in vitro	Infection	[94]
2020	N	Collagen	Sponge	Nitrosothiol	-	-	-	-	in vivo	-	[95]
2019	S	PEI	Hydrogel	NONOate	Yes	-	-	-	in vivo	-	[96]
2019	S	PAA	Hydrogel	Sildenafil citrate	-	-	-	-	in vivo, in vitro	-	[97]
2019	S	PEI, PLGA	np	NONOate	Yes	-	-	Yes	in vivo, ex vivo, in vitro	Diabetic infection	[98]
2018	S	PLGA	Emulsion	Nitrosothiol	-	-	-	Yes	in vivo, in vitro	Infection	[99]
2017	SS	PCL/chitosan	Hydrogel	NONOate	Yes	-	Enzyme	-	in vivo	Ischemia	[35]
2016	S	Poly-siloxane	Hydrogel	NONOate NONOate	Yes	-	-	Yes	Clinical	Acne lesion	[100]
2015	S	PVA/PL	Hydrogel	Nitrosothiol	Yes	-	-	-	in vivo	-	[101]
2015	S	PAN	Hydrogel	Acrylonitrile	Yes	-	-	-	in vivo	-	[102]
2015	N	Chitosan	Hydrogel	Nitrosothiol	-	-	-	Yes	in vivo, in vitro	-	[103]
2013	N	Nap-FFGGG	Hydrogel	NONOate	Yes	-	Enzyme	-	in vivo	-	[104]
2012	SS	Alginate/Acrylic polymer	Chamber	Inorganic nitrite	-	Probiotics	-	Yes	in vivo, in vitro	Ischemic, infection	[105]
2010	S	PL	Hydrogel	Nitrosothiol	-	-	-	-	in vivo	Ischemia	[106]
2009	S	PEG/Disaccharide	Hydrogel	Inorganic nitrite	-	-	-	Yes	in vivo, in vitro	Infection	[107]
2008	S	PL	Hydrogel	Nitrosothiol	-	-	-	-	in vivo	-	[108]
2007	S	PL	Hydrogel	Nitrosothiol	-	-	-	-	in vivo	-	[109]
2002	S	PVA	Hydrogel	NO• gas	Yes	-	-	-	in vivo	Diabetes	[110]

Abbreviations. Polymer origin: natural (N), semi synthetic (SS), synthetic (S). Polymer carriers: poly(lactic-co-glycolic acid) (PLGA), poly(ethylene glycol) (PEG), polyurethane (PU), gelatin methacrylamide (gelMA), poly(ethylene imine) (PEI), poly(acrylic acid) (PAA), poly(ε-caprolactone) (PCL), poly(vinyl alcohol) (PVA), Pluronic F-127 (PL), poly(acrylonitrile) (PAN), naphthalene-capped amino acids (phenylalanine/glycine 2:3) (Nap-FFGGG). Matrix: nanoparticles (np). NO-donor: diazeniumdiolate (NONOate).

## Data Availability

Not applicable.
